# Gut proteases target *Yersinia *invasin *in vivo*

**DOI:** 10.1186/1756-0500-4-129

**Published:** 2011-04-18

**Authors:** Janja Trček, Marc F Oellerich, Katy Niedung, Frank Ebel, Sandra Freund, Konrad Trülzsch

**Affiliations:** 1 Max von Pettenkofer Institut für Hygiene und Medizinische Mikrobiologie, Ludwig Maximilians Universität München, Germany

## Abstract

**Background:**

*Yersinia enterocolitica *is a common cause of food borne gastrointestinal disease. After oral uptake, yersiniae invade Peyer's patches of the distal ileum. This is accomplished by the binding of the *Yersinia *invasin to β1 integrins on the apical surface of M cells which overlie follicle associated lymphoid tissue. The gut represents a barrier that severely limits yersiniae from reaching deeper tissues such as Peyer's patches. We wondered if gut protease attack on invasion factors could contribute to the low number of yersiniae invading Peyer's patches.

**Findings:**

Here we show that invasin is rapidly degraded *in vivo *by gut proteases in the mouse infection model. *In vivo *proteolytic degradation is due to proteolysis by several gut proteases such as trypsin, α-chymotrypsin, pancreatic elastase, and pepsin. Protease treated yersiniae are shown to be less invasive in a cell culture model. YadA, another surface adhesin is cleaved by similar concentrations of gut proteases but Myf was not cleaved, showing that not all surface proteins are equally susceptible to degradation by gut proteases.

**Conclusions:**

We demonstrate that gut proteases target important *Yersinia *virulence factors such as invasin and YadA *in vivo*. Since invasin is completely degraded within 2-3 h after reaching the small intestine of mice, it is no longer available to mediate invasion of Peyer's patches.

## Background

*Y. enterocolitica *and *Y. pseudotuberculosis *cause food-borne gastrointestinal disease in humans. While *Y. pseudotuberculosis *is primarily an animal pathogen that only rarely causes disease in humans, *Y. enterocolitica *is one of the most common causes of gastroenteritis especially in northern Europe where *Y. enterocolitica *is the third most common cause of bacterial gastroenteritis [1]. *Yersinia *infection can manifest as enteritis, terminal ileitis, or mesenteric lymphadenitis (pseudoappendicitis) but can occasionally lead to septicaemia with abscess formation in liver and spleen in predisposed patients (e.g. iron overload). After several weeks, immunological sequelae such as reactive arthritis or erythema nodosum may complicate yersiniosis [1]. After oral uptake, yersiniae are known to invade Peyer's patches (PP) of the ileum by entering through specialized epithelial cells called M cells. This is made possible by the interaction of the *Yersinia *invasin with β1 integrins which are expressed on the luminal side of M cells but not enterocytes [2]. After translocation across the mucosal barrier, yersiniae subsequently disseminate to lymph nodes, spleen and liver where they form monoclonal microabscesses [3].

Invasin (Inv) is a chromosomally encoded outer membrane protein which is expressed at low temperature and early stationary phase, conditions which are prevalent in stored foods [4,5]. Invasin is anchored by its N-terminal region in the outer membrane. In *Y. pseudotuberculosis *the C-terminal end forms an 18 angstrom rod consisting of five globular domains (D1-D5) [6]. The C-terminal cell adhesion superdomain (D4 and D5) is the minimal region that is able to bind αβ1 integrins [7,8]. High affinity binding by *Y. pseudotuberculosis *however also requires the D2 domain, which is absent in *Y. enterocolitica*. D2 mediates oligomerization of Inv on the bacterial surface which promotes the clustering of integrin receptors on host cell membrane generating a critical signal that leads to internalization of bacteria [9].

Another adhesin besides Inv, which is able to potentially interact with β1 integrins of M cells is the pYV plasmid encoded *yadA*, the prototype of a novel class of nonfimbrial adhesins (the oligomeric coiled coil adhesin family) [10,11]. YadA is a 41-44 kDa surface protein that forms trimers in the outer membrane. These appear as 23 nm lollipop-shaped projections consisting of an 18 nm stalk and a 5 nm head [12]. YadA binds diverse extracellular matrix (ECM) proteins such as collagen, fibronectin and laminin [13,14]. Furthermore YadA mediates indirect binding of yersiniae to β1 integrins through fibronectin-β1 integrin bridging [15,16]. As is the case for the Inv-β1 integrin interaction, the YadA-fibronectin-β1 integrin bridging leads to IL-8 production and to internalization of yersiniae by Hep-2 cells. These effects are however short lived and are weaker than for Inv [16]. YadA is a multifunctional protein [14] that is responsible not only for adhesion and bacterial autoagglutination but also protects yersiniae from the actions of defensins and confers resistance to serum complement lysis. Serum resistance is mediated by the stalk domain whereas adherence to neutrophils and collagen binding are mediated by the head region. A *yadA *knock out mutant was highly attenuated both after oral and i.v.- infection [17].

Invasin is the most important mediator of invasion into Peyer's patches *in vivo *but YadA and the attachment-invasion locus protein (Ail) which mediates attachment and invasion of eukaryotic cells *in vitro *are further candidates that might play a role *in vivo *[18,19]. The chromosomally encoded *myf *(mucoid *Yersinia *factor) is similar to the pH 6 antigen of *Y. pestis *and is composed of 15 kDa subunits that appear as a fibrillar layer surrounding bacteria. Myf enhances binding to cells and intestinal mucus [20]. We have recently discovered that only very few yersiniae of a large inoculum (10^9^) invade Peyer's patches from the gut lumen in the mouse infection model [3]. We are now exploring mechanisms that lead to this phenomenon. Since Inv, YadA, and Myf are exposed on the bacterial surface, they could be targets of gut proteases such as trypsin, α-chymotrypsin, pancreatic elastase, carboxypeptidases or pepsin. In fact Inv has been shown to be cleaved by extracellular trypsin *in vitro *[21,22]. Proteolytic degradation of these proteins in the intestine would obviously limit contact of *Yersinia *with M cells of the distal ileum contributing to clonal invasion. Although it has been shown that pathogenic bacteria are targeted by stomach acid and defensins [23-26] in the GI tract, there are no reports of the effects of gut proteases on virulence factors of enteric pathogens.

## Methods

### Bacterial strains and plasmids

*Y. enterocolitica *WA-314 is a clinical isolate of serotype O:8 [27]. WA-C is a derivative of WA-314 [27] not harboring the pYV plasmid [3]. WA-C(pYV-kan) harbors a kanamycin cassette in a non-coding region of the pYV plasmid [3]. WA-C-inv and WA-314-inv are isogenic invasin mutants of WA-C and WA-314 respectively [28]. To construct a *myfA *mutant, a 645 bp *myf*-fragment was subcloned and insertional inactivation of the *myfA *gene was accomplished by introducing a spectinomycin resistance cassette amplified from Ω plasmid into the internal KpnI restriction site of the *myfA *gene. The *myfA::spec *construct was transferred to the suicide plasmid pEP185.2. This resulting mutator plasmid pEP185.2*myfA::spec *was mobilized into strain WA-314 by conjugation from *E. coli *S17-1λpir, and the chromosomal *myfA *gene was replaced with the *myfA::spec *construct by double recombination. Allelic exchange was confirmed by PCR. Bacteria were cultured aerobically in Luria-Bertani (LB) broth or on LB agar plates at 27°C. Antibiotics were used at the following concentrations (μg/ml); kanamycin: 25; chloramphenicol: 20, tetracycline: 20.

### Oral mouse infection

**Figure 1:**
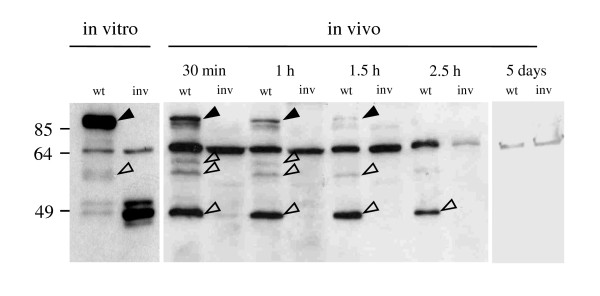
**Proteolytic degradation of invasin in mouse small intestine**. 5 Balb/c mice were infected orally with 10^9 ^CFU *Y. enterocolitica *WA-314 (wt) or an isogenic *inv *mutant (inv). Mice were sacrificed at the indicated time points and bacteria were washed from small intestinal lumen with PBS. For each lane 3 × 10^6 ^yersiniae were subjected to Western blotting with an a Inv polyclonal antibody. Solid arrowheads point to full length Inv, arrowheads point to specific Inv degradation products.

6-8 week old female Balb/c mice (Harlan-Winkelmann) were kept under specific pathogen-free-conditions (positive-pressure cabinet) and were provided with food and water *ad libitum*. Mice were infected orally with yersiniae grown to stationary phase in LB medium at 27°C. Mice were sacrificed by CO_2 _asphyxiation and small intestines were aseptically removed. Bacteria were washed from the small intestines (SI) with PBS, and filtered with a 5 μm Durapore filter (Millipore) to remove debris. The number of yersiniae in the lavage was determined by plating serial dilutions on CIN agar. The indicated number of yersiniae were subjected to SDS PAGE on 11.5% polyacrylamide gel (Figure 1). Immunoblotting was performed using nitrocellulose sheets. Blocking was performed with 5% BSA in PBS overnight at 4°C. Polyclonal rabbit anti-Inv antiserum recognizing the 4 C-terminal domains of *Y. enterocolitica *invasin and a horseradish-peroxidase-conjugated secondary anti-rabbit antibody were used for immunostaining. The rabbit α Inv antibody was raised against a recombinant protein containing the extracellular domains D1, D3, D4, and D5 (C-terminal 397 AA) of *Y. enterocolitica *WA-314. This region harbours the β1 integrin binding site but not the membrane anchor. All mouse experiments were approved by government authorities (Regierung von Oberbayern, AZ 55.2-1-54-2531-153-07).

### Adhesion and Invasion assays

HeLa cells were infected with yersiniae grown at 27°C at MOIs of 50 in 24 well plates. Yersiniae were centrifuged onto cells and incubated at 37°C for 90 min. Extracellular yersiniae were killed by addition of gentamicin (50 μg/ml) for 1 h. Cell lysis was performed with 0.1% triton/PBS. The number of intracellular and extracellular bacteria was determined by plating serial dilutions of bacteria on agar plates as described previously [29]. Adhesion assays were performed at 20°C, invasion experiments at 37°C. Results are expressed as percent invasion (100 × CFU of *Yersinia *resistant to gentamicin/cell associated bacteria). Plating serial dilutions of protease treated yersiniae demonstrated viability of yersiniae after protease treatment. Bacteria treated with proteases were not found to be hypersensitive to the Triton detergent. Statistical analysis was performed using Mann-Whitney test at the 0.05 significance level.

### Protease digests

**Figure 2:**
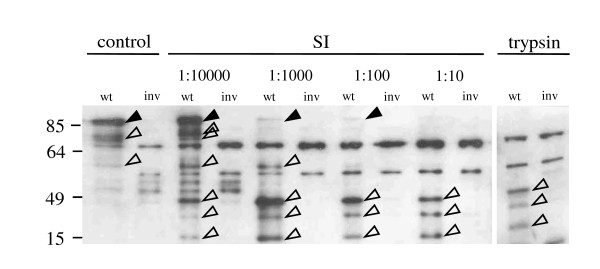
***In vitro *proteolytic degradation of invasin**. *Y. enterocolitica *WA-314 (wt) or the invasin mutant (inv) were digested for 2 h at 37°C with small intestinal fluid of mice (SI) in different dilutions (1:10 - 1:10000). Digests with trypsin and trypsin digestion buffer (control) are also shown. The small intestines of mice were washed with 2 ml trypsin digestion buffer, centrifuged, and the supernatant was used to digests 3 × 10^6 ^CFU of yersiniae. Immunoblotting was performed with an a inv polyclonal antibody. Solid arrowheads point to full length Inv, open arrowheads point to specific Inv degradation products.

**Figure 3:**
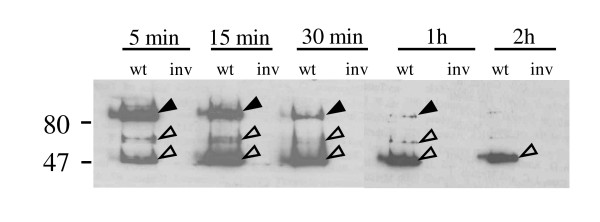
**Kinetics of proteolysis by human ileal fluid**. 10^8 ^CFU *Y. enterocolitica *WA-314 (wt) or the invasin mutant (inv) were digested at 37°C for the indicated time with 1 ml human ileal fluid. For each lane 3 × 10^6 ^yersiniae were subjected to Westerm blotting with an a Inv polyclonal AB. Solid arrowheads point to full length Inv, open arrowheads point to specific Inv degradation products.

**Figure 4:**
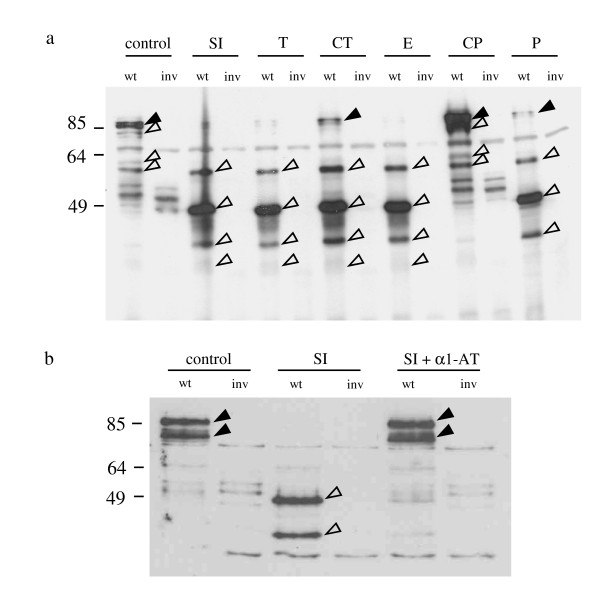
**Serine proteases target invasin**. (a) Wild-type *Y. enterocolitica *(wt) or the invasin mutant (inv) were digested with small intestinal fluid from mice (SI), α-chymotrypsin (CT), trypsin (T), carboxypeptidase A (CP), pepsin (P), elastase (E) or trypsin digestion buffer (control) for 2 h at 37°C. SI was washed with 2 ml PBS, centrifuged, and supernatant was used for digests. 1 × 10^7 ^CFU of yersiniae were subjected to Western blotting with an a inv polyclonal AB. (b) *Y. enterocolitica *WA-314 (wt) or the invasin mutant (inv) were digested with the supernatant of small intestinal luminal fluid (SI), mock digested with PBS or digested with SI preincubated with a1-antitrypsin for 1 h at 37°C. 1 × 10^7 ^CFU of yersiniae were subjected to Western blotting with an α inv polyclonal AB. Solid arrowheads point to full length Inv, open arrowheads point to specific Inv degradation products.

**Figure 5:**
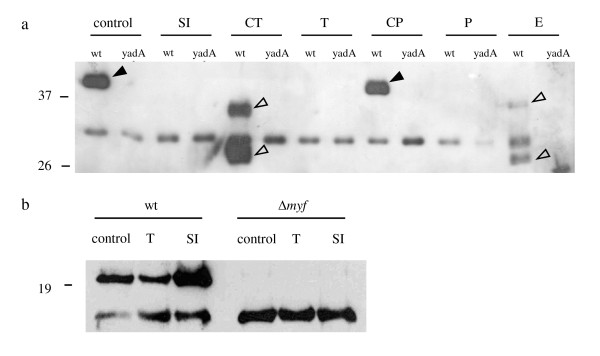
**YadA but not Myf is degraded by gut proteases**. *Y. enterocolitica *WA-314 (wt), *yadA *mutant (yadA) or Δ*myf *mutant were digested with small intestinal (SI) luminal fluid, α-chymotrypsin (CT), trypsin (T), carboxypeptidase A (CP), pepsin (P), elastase (E) or trypsin digestion buffer (control) for 2 h at 37°C. SI was washed with 2 ml PBS centrifuged and supernatant was used for digests. 1 × 10^7 ^CFU yersiniae were subjected to Western blotting with a polyclonal YadA (a) or Myf (b) antibody. Solid arrowheads point to full length YadA, open arrowheads point to specific YadA degradation products.

**Figure 6:**
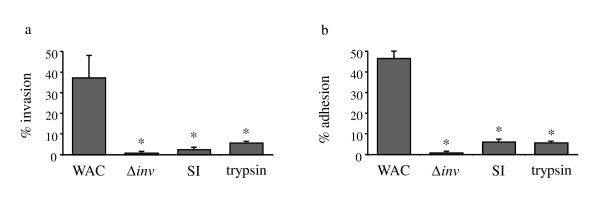
**Gut protease treated yersiniae are less invasive *in vitro***. *Y. enterocolitica *WA-C or the invasin mutant WA-C-inv (Δ*inv*) were digested with the supernatant of small intestinal luminal fluid (SI) or trypsin or mock digested with trypsin digestion buffer. Invasion (a) and adhesion (b) assays were performed using HeLa cells. Invasion assay was performed at 37°C, adhesion assay at 20°C. Percent yersiniae surviving gentamicin treatment is shown in (a), Percent yersiniae adhering to cells and SD is shown in (b). Statistical analysis was performed using Mann-Whitney test at the 0.05 significance level. Asteriks indicate a significant difference from WA-C.

Yersiniae in Figure 2 lanes 11 and 12 were digested with 2,5 mg/ml trypsin in Tris-base pH 8.0, 11.5 mM CaCl_2_. This is higher than concentrations of trypsin found in intestinal fluid of humans [30]. Yersiniae in Figure 3 were digested with undiluted human ileal fluid. Yersiniae in Figures 4, 5 and 6 were digested with trypsin (23 μg/ml in Tris-base pH 8.0, 11.5 mM CaCl_2_), α-chymotrypsin (20 μg/ml in 100 mM Tris/Cl pH 7.8, 10 mM CaCl_2_), pancreatic elastase (10 μg/ml 100 mM NH_4_HCO_3 _pH 8.0) or carboxypeptidase A (50 μg/ml in 25 mM Tris/Cl pH 7.65). These concentrations are below physiological values which have been reported to be about 280 μg/g feces, 450 μg/g feces, and 200 μg/g feces respectively [30]. The pepsin digest in Figure 4a (4 mg/ml in 10 mM HCl) was performed using a concentration slightly higher than that reported for human gastric juice of 0.5 -1 mg/ml [31]. All proteases were obtained from Sigma-Aldrich.

10^7 ^yersiniae grown at 27°C in LB medium were washed with PBS and digested at 37°C or 27°C in the respective buffers. Following digestion for 1 h yersiniae were harvested by centrifugation and subjected to immunoblotting as described above. Yersiniae were also digested with small intestinal secretions of Balb/c mice. For this purpose, SI was washed with 3 ml PBS, centrifuged and the supernatant was used for digests. Protease inhibition was performed with α1 antitrypsin (10 mg/ml).

## Results

### Inv is degraded *in vivo *by gut proteases

To determine if the surface exposed *Yersinia *virulence factor invasin (Inv) is targeted by gut proteases in the mouse infection model, we orally infected groups of five Balb/c mice with 10^9 ^CFU *Y. enterocolitica *wild type and an isogenic *inv *mutant. Bacteria were washed from the lumen of the small intestine after 30 min, 1 h, 1.5 h, and 2.5 h post infection and were subjected to Western blotting using a polyclonal invasin antibody. As can be seen in Figure 1 the 92 kDa full length Inv protein is rapidly degraded *in vivo*. Only 30 min after oral infection of mice a significant amount of invasin is degraded to a 49 kDa protein with only this degradation product and no full length Inv remaining after 2.5 h. Several larger partial degradation products (open arrowheads) can also bee seen in yersiniae cultured in vitro in the absence of protease treatment (lane 1 of Figure 1, 2, 3 and 4). This is a well known phenomenon that has been described previously [21,22]. To determine if Inv is subsequently produced in the gut lumen, we performed immunoblotting using the Inv antibody on yersiniae washed from the SI of mice that were infected for 5 days. We could not detect full length protein or degraded Inv by Western blotting (Figure 1). These results indicate that the major invasion factor of *Yersinia *is degraded within a few hours after oral uptake by gut proteases and is subsequently not produced in the gut lumen at detectable levels. This presumably limits Inv mediated early invasion of PPs by *Yersinia *to a very short time window of a few hours only.

### Inv is cleaved *in vitro *into 3 peptides

To determine if *in vivo *degradation could be reproduced *in vitro*, we digested wild-type yersiniae grown in LB medium at 27°C with serial dilutions (1:10-1:10000) of SI fluid and trypsin for 2 h at 37°C (Figure 2). As a control, digests were performed with the *inv *mutant. This experiment revealed that three distinct Inv degradation products were generated by *in vitro *digestion of yersiniae with either concentrated or a 1:10 dilution of SI luminal secretions. Complete digestion with a high trypsin concentration revealed an identical peptide pattern. Even when SI fluid was highly diluted (1:1000 - 1:10000), Inv was degraded but larger partial degradation products were also visible. This shows that Inv is highly susceptible to gut protease cleavage even at concentrations below physiological levels.

Interestingly several non-specific peptides (seen also for the *inv *mutant in Figure 2) were also degraded by gut proteases. To determine if the *in vivo *observed kinetics of digestion could be reproduced *in vitro*, undiluted human ileal secretions obtained by endoscopy from patients were used to digest yersiniae grown in LB medium. This experiment showed that full length Inv was degraded to a 49 kDa peptide within about 2 h (Figure 3) as was the case in the *in vivo *mouse experiment.

### Serine proteases target invasion

To identify gut proteases that might be responsible for the observed Inv degradation *in vivo*, we performed protease digests with *in vitro *grown yersiniae using trypsin, α-chymotrypsin, pancreatic elastase, carboxypeptidase A and pepsin. For this purpose 10^7 ^yersiniae were grown at 27°C in LB medium, washed with PBS and digested in their respective buffers for 2 h. Yersiniae were harvested by centrifugation and subjected to immunoblotting with a polyclonal anti-Inv antiserum. In parallel yersiniae were digested with small intestinal secretions of Balb/c mice. These experiments showed that invasin is degraded to a major 49 kDa peptide non-specifically by multiple proteases such as trypsin, α-chymotrypsin, elastase, and pepsin, but not by carboxypeptidase A (Figure 4a). Digests with carboxypeptidase showed only 3 specific larger partial degradation products (ca. 60-80 kDa) that are also seen in the non digested control yersiniae. The slightly differing band pattern seen in Figure 4a vs. Figure 2 is due to incomplete digestion of the 55 kDa peptide seen in Figure 4. These partial digestion products can also be seen in lanes 3 and 5 of Figure 2 when highly diluted (1:1000 and 1:10000) SI secretions from mice were used for digests.

To determine if protease cleavage in the SI was due to serine proteases, digests of yersiniae with SI secretions were performed in the presence of α1 antitrypsin. These experiments showed that degradation of Inv by SI secretions could be inhibited by a serine protease inhibitor. As can be seen in Figure 4b, full length Inv and a slightly smaller spontaneous degradation product are completely digested to the 49 and 25 kDa peptides only in the absence of protease inhibitor. To determine if there were differences in susceptibility to different proteases, digests were performed with trypsin, α-chymotrypsin, and elastase at concentrations between 0.001 μM - 10 μM. This experiment revealed that Inv was most susceptible to trypsin cleavage being completely degraded in 1 h at a concentration of 0.1 μM followed by α-chymotrypsin and elastase which are completely degraded at a concentration of 1 μM (results not shown).

### YadA but not Myf is degraded by gut proteases

To determine if protease attack on Inv was a non-specific phenomenon, other surface proteins were studied by Western blotting after treating yersiniae with SI secretions or proteases (Figure 5). These experiments revealed that YadA is also cleaved quite non-specifically by several gut proteases such as trypsin α-chymotrypsin, pepsin and elastase (but not carboxypeptidase A) at concentrations similar to those that cleave Inv. SI secretions from mice as well as trypsin and pepsin completely degraded YadA, whereas α-chymotrypsin and elastase digests revealed 2 distinct degradation products (open arrowheads in Figure 5). In contrast to YadA and Inv however, Myf was not degraded by SI secretions or trypsin, demonstrating that not all surface proteins are degraded unspecifically in the gut.

### Protease treated yersiniae are less invasive *in vitro*

To determine if gut protease attack on *Yersinia *is relevant for the infection process, we performed adhesion and invasion assays using HeLa cells. For these experiments yersiniae lacking the virulence plasmid pYV were digested with SI secretions or trypsin (0.23 μg/ml). Viability of yersiniae after protease treatment was not compromised and verified by plating serial dilutions of protease treated yersiniae. Tetracycline was added to inhibit protein synthesis during cell culture experiments (to inhibit new Inv expression). Yersiniae treated with protease digestion buffer were used as controls. These experiments revealed that yersiniae pre-treated with SI secretions or trypsin were much less invasive (similar to an *inv *mutant) than yersiniae treated with trypsin digestion buffer. Furthermore protease digested yersiniae were much less adherent to HeLa cells than control bacteria (Figure 6) as expected.

## Discussion

While passing through the GI tract enteropathogenic bacteria are subjected to many adverse conditions such as the acidity of the stomach, antimicrobial peptides and gut proteases. The relevance of most of these factors in killing or inhibiting bacteria has been demonstrated [23,24,32]. However there are no reports of the effects of gut proteases on surface exposed virulence factors of enteric pathogens. We therefore studied the role of gut proteases on surface exposed virulence factors of *Yersinia *such as Inv, YadA, and Myf.

It has previously been demonstrated that only very few yersiniae invade Peyer's patches from the gut lumen resulting in the formation of very few monoclonal microabscesses [3,33]. The host has been shown to severely restrict the sequential invasion of Peyer's patches by yersiniae [3] which obviously contributes to the low number of *yersiniae *invading and abscessing PPs. But the low frequency with which *Yersinia *initially establishes abscesses in PPs cannot be explained by this mechanism. Since Inv is a surface located protein we postulated that Inv is attacked by gut proteases in the small intestine which would prevent interaction of yersiniae with β-integrins of M cells. To investigate the fate of invasin at very early time-points after infection, we orally infected mice with *Y. enterocolitica *and analysed Inv production by Western blotting in the small intestines of mice. The 92 kDa full length Inv protein of yersiniae grown at 27°C was still detectable in the small intestine shortly after oral infection but was rapidly degraded *in vivo*. Only 30 min after oral infection a significant amount of invasin was degraded to a ca. 49 kDa protein with only this degradation product and no full length Inv remaining after 2.5 h. As reported previously, Inv was not detectable in the SI lumen between 2 and 5 days after oral infection [3]. These results indicate that this major invasion factor of *Yersinia *is degraded within a few hours after oral uptake by gut proteases and is no longer available to mediate invasion of PPs. Of course other factors such as pH, gut-microbiota and other gut-environmental factors could also cause similar effects. Not surprisingly the invasion capacity of yersiniae for epithelial cells was greatly reduced when they were treated with trypsin or SI fluid prior to infection of HeLa cells. There is currently no suitable cell culture model to study invasion of M-cells *in vitro *and HeLa cells may not necessarily represent a suitable model for M cell invasion. Therefore it will be interesting to study the effects of gut proteases on the invasion of PPs *in vivo*. It is of course possible that *in vivo *yersiniae might be protected from the action of intestinal proteases in certain niches. They could for example be protected by intestinal mucus or might produce a biofilm in the gut. Possibly Inv could be protected from digestion by other *Yersinia *surface proteins such as YadA or Myf.

Proteolytic cleavage of Inv *in vitro *showed 3 distinct degradation products that were associated with yersiniae. We attempted to identify these degradation products in membrane preparations of *Yersinia *by MALDI-TOF but this was not possible since digested Inv peptides were not released into the supernatant and membrane preparations of *Yersinia *contained many proteins of similar size. We were, however, successful in identifying cleavage products by MALDI-TOF and protein sequencing using a recombinant protein consisting of 397 C terminal amino acids of Inv [34]. However, these peptides were distinct from those that were generated by digesting whole bacteria. Nevertheless, since the Inv antibody that we used is directed against domains D1- D5 of Inv, the major degradation product of about 49 kDA that was observed in yersiniae washed from the SI of mice must represent the Inv membrane anchor with part of the D1 domain still intact.

Besides Inv we demonstrated that other surface proteins of *Yersinia *such as YadA are also readily degraded by gut proteases. However, protease attack on surface proteins does not seem to be a general phenomenon since MyfA which forms fibrillae on the *Yersinia *surface extending 2 microns [20] is not degraded by similar concentrations of trypsin or small intestinal fluid.

Gut proteases could have several effects on invasins and adhesins of bacteria. It is possible that such factors are activated by proteolysis. This has been shown for reovirus where luminal proteolytic digestion was important for rendering progeny virions infectious in the gut [35]. Alternatively, proteolysis of such adhesion factors might lead to inactivation and protect the host from hyperinvasion by *Yersinia*. Furthermore, proteolysis of invasins and adhesins could promote bacterial spreading to new hosts by increasing shedding of yersiniae in feces.

## Conclusions

*Yersinia *virulence factors such as invasin are targeted by gut proteases in the lumen of the small intestine. Invasin, the most important invasion factor for Peyer's patches is rapidly degraded in the small intestine within 2-3 h after oral infection of mice and is therefore no longer available to mediate invasion of Peyer's patches. This is consistent with the finding that *Yersinia *invasion occurs very early during the infection process and might contribute to the low numbers of yersiniae reaching Peyer's patches after oral infection of mice.

## Competing interests

The authors declare that they have no competing interests.

## Authors' contributions

JT, MFO, KN, FE, KT performed experiments. KT conceived and designed the study. JT, and KT analyzed the data and wrote the manuscript. All authors approved the final manuscript.
